# Recapitulation of Neural Crest Specification and EMT via Induction from Neural Plate Border-like Cells

**DOI:** 10.1016/j.stemcr.2020.07.023

**Published:** 2020-08-27

**Authors:** Gerson Shigeru Kobayashi, Camila Manso Musso, Danielle de Paula Moreira, Giovanna Pontillo-Guimarães, Gabriella Shih Ping Hsia, Luiz Carlos Caires-Júnior, Ernesto Goulart, Maria Rita Passos-Bueno

**Affiliations:** 1 Centro de Pesquisa sobre o Genoma Humano e Células-Tronco, Departamento de Genética e Biologia Evolutiva, Instituto de Biociências, Universidade de São Paulo, São Paulo, Brazil

**Keywords:** epithelial-to-mesenchymal transition, pluripotent stem cells, disease modeling, cell fate, neurocristopathy, transdifferentiation

## Abstract

Neural crest cells (NCCs) contribute to several tissues during embryonic development. NCC formation depends on activation of tightly regulated molecular programs at the neural plate border (NPB) region, which initiate NCC specification and epithelial-to-mesenchymal transition (EMT). Although several approaches to investigate NCCs have been devised, these early events of NCC formation remain largely unknown in humans, and currently available cellular models have not investigated EMT. Here, we report that the E6 neural induction protocol converts human induced pluripotent stem cells into NPB-like cells (NBCs), from which NCCs can be efficiently derived. NBC-to-NCC induction recapitulates gene expression dynamics associated with NCC specification and EMT, including downregulation of NPB factors and upregulation of NCC specifiers, coupled with other EMT-associated cell-state changes, such as cadherin modulation and activation of TWIST1 and other EMT inducers. This strategy will be useful in future basic or translational research focusing on these early steps of NCC formation.

## Highlights

• Human neural plate border-like cells (NBCs) emerge during the E6 neural induction
• NBC-to-NCC induction recapitulates sequential steps of NCC specification and EMT
• This approach provides a human cell model of early NCC development

## Teaser

Kobayashi et al. show that neural conversion of hiPSCs produces NPB-like cells that can be efficiently converted into NCCs. This enables observation and recapitulation of sequential molecular and cellular events involved in NC specification and EMT, and provides an *in vitro* platform for investigating human early NCC development and associated disorders.

## Introduction

Neural crest cells (NCCs) are a multipotent cell population that plays pivotal roles in vertebrate embryonic development. In late-blastula stages, intermediate BMP levels specify cells residing between the prospective neural and non-neural ectoderm into the neural plate border (NPB), a broad competence domain that contributes to formation of the neural tube, neural crest (NC), and the preplacodal and non-neural ectoderm (Pla and Monsoro-Burq, 2018). Definitive cell fates are not yet established in the NPB, which expresses ectodermal transcription factors (called NPB specifiers) associated with development of either the neural or the non-neural ectoderm, and they are required for segregation of the NC territory (Maharana and Schlosser, 2018; Roellig et al., 2017). In the NPB, inductive signals that include the WNT and BMP pathways activate a complex network of transcription factors that regulate NC specification and epithelial-to-mesenchymal transition (EMT), a series of molecular events that transitions cells from an epithelial to a mesenchymal, migratory phenotype. After EMT, NCCs migrate through the cranial and trunk regions of the embryo, contributing to the morphogenesis of several cells and tissues, such as craniofacial cartilage and bones, melanocytes, and Schwann cells (Theveneau and Mayor, 2012).

Disturbances in any of the above steps may impair NC development and result in several human health conditions. These include congenital disorders arising from defects in NC derivatives, such as craniofacial syndromes, pigmentation disorders, Hirschprung disease, and others, and also aggressive cancers of NC origin, such as neuroblastoma and melanoma (Vega-Lopez et al., 2018). Due to limited access to human embryonic tissues, knowledge on the pathophysiological mechanisms behind these disorders has been largely derived from animal models, which show discrepancies across species and may not completely recapitulate human embryonic development (Barriga et al., 2015; Thyagarajan et al., 2003). In addition, analysis of early events of NC formation, such as specification and EMT, is difficult to perform in mice, the main model organism of mammalian NC biology (Prasad et al., 2019). Thus, gaining better insight into human NC formation requires development of alternative approaches, such as stem cell-based models. There are many elegant reports characterizing NCC generation from human induced pluripotent stem cells (hiPSCs) or embryonic stem cells (Hackland et al., 2017; Lee et al., 2010; Leung et al., 2016; Liu et al., 2012; Menendez et al., 2013; Miller et al., 2017; Tchieu et al., 2017; Thier et al., 2019). Despite providing deeper understanding into some disorders, these strategies have been largely applied to investigate late phases of NC development, such as migration and differentiation (Srinivasan and Toh, 2019). Moreover, most of them do not include an NPB stage, and although EMT has been extensively modeled in human cells in the context of cancer (Yang et al., 2020), currently there is still no *in vitro* approach describing human NC EMT.

Here, we report a simple hiPSC-based procedure to investigate early NC formation. This procedure recapitulates gene expression dynamics and other cellular hallmarks associated with embryonic NPB cells transitioning from a neuroepithelial to an NC mesenchymal state, allowing for time series assessment of key molecular events orchestrating NC specification and EMT. This approach could be used to model human NC-related disorders, particularly those associated with these early events of NC development.

## Results

### Characterization of the E6 Neural Induction

**Figure 1:**
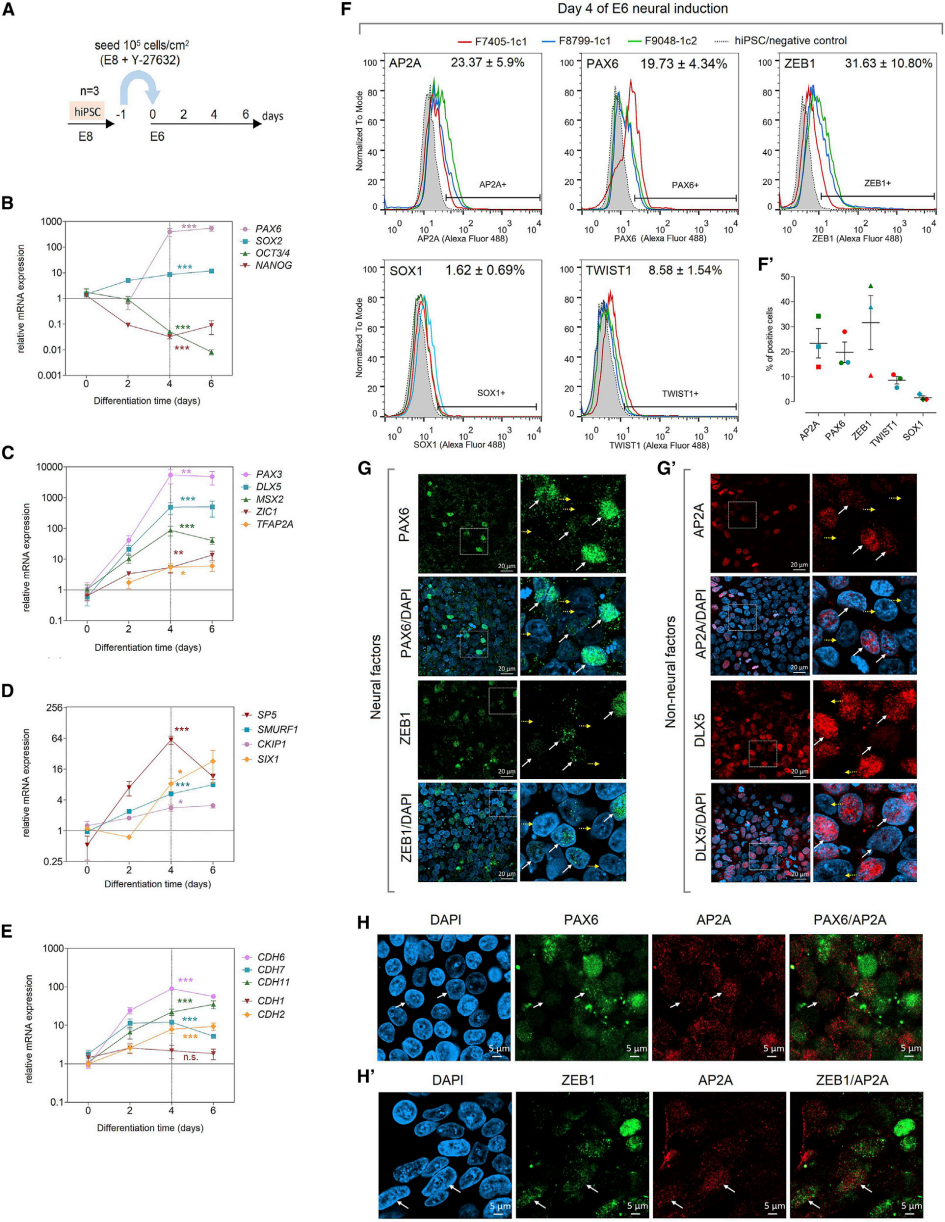
Neural Induction with the E6 Method Produces NPB-like Cells in 4 Days (A) Schematic representation of the E6 induction. (B) Assessment of neural conversion via qRT-PCR for neural fate markers *SOX2* and *PAX6*, and pluripotency markers *OCT3/4* and *NANOG*. (C and D) qRT-PCR for key NPB specifiers *PAX3*, *DLX5*, *MSX2*, *ZIC1*, and *TFAP2A* (C) and additional NPB factors *SP5*, *SMURF1*, *CKIP1*, and *SIX1* (D) during E6 induction. (E) qRT-PCR for cadherins involved in NC development *CDH1*, *CDH2*, *CDH6*, *CDH7*, and *CDH11*. qRT-PCR data are plotted as mean ± SEM (n = 3 biological replicates). ^∗∗∗^p < 0.001, ^∗∗^p < 0.01, ^∗^p < 0.05; ns, not significant; one-way ANOVA with Dunnett's correction. (F and F′) Flow cytometry analysis on day 4 of induction for AP2A, PAX6, ZEB1, TWIST1, and SOX1; values for each sample are plotted in (Fʹ). Bars represent mean ± SEM (n = 3 biological replicates). (G and G′) Representative immunostaining for neural factors PAX6 and ZEB1 (G), and for non-neural factors AP2A and DLX5 (G′) on day 4 of induction (n = 3 biological replicates). White arrows show variable nuclear staining intensity; yellow arrows point to negative nuclei. Right panels are higher-magnification images. Scale bar, 20 μm. (H and H′) Representative double immunostaining for AP2A/PAX6 (H) and AP2A/ZEB1 (H′) on day 4 of induction (n = 3 biological replicates). Arrows point to double-positive nuclei. Scale bar, 5 μm. Related to Figure S1.

First, we selected a neural induction method as a starting point for NC derivation. The “E6 method” generates neuroepithelial cells with the Essential 6 (E6) minimal medium, without blocking the BMP pathway, in 6 days (Lippmann et al., 2014). Three hiPSC lines (F7405-1c1, F8799-1c1, and F9048-1c2) were grown in Essential 8 (E8) medium, and then subjected to the E6 neural induction (Figure 1A). During induction, pluripotency markers *OCT3/4* and *NANOG* were downregulated, while neural markers *SOX2* and *PAX6* showed increased mRNA expression on day 4, persisting until day 6 (Figure 1B). On day 4, mesoderm specifier *T* and endoderm specifier *SOX17* exhibited minimal expression signals similar to hiPSCs (Figure S1A). After 6 days of induction, PAX6 was expressed in nuclei, while Musashi-1, a neural stem cell marker (MacNicol et al., 2015), was localized to the cytoplasm, and N-cadherin (CDH2) was localized to plasma membranes at cell-cell contact sites (Figure S1B). Finally, flow cytometry analysis of day 6 cells showed positive staining (46.67%) for SOX1, a neural specifier co-expressed with PAX6 in the human neural tube (Zhang et al., 2010) (Figure S1C). These results confirm that neural identity is attained on day 6 of E6 neural induction.

### An NPB-like Identity Emerges on Day 4 of E6 Neural Induction

In comparison with day 0, cells on day 4 of induction showed increased mRNA expression of neural factors *PAX6* and *SOX2* (Figure 1B), which are also expressed in embryonic NPB cells (Roellig et al., 2017; Thier et al., 2019). Therefore, we investigated several additional factors involved in NPB formation during the 6 days of neural induction. We found that key NPB specifiers *TFAP2A*, *DLX5*, *PAX3*, *ZIC1*, and *MSX2* were also upregulated on day 4 (Figure 1C), along with other NPB factors *SP5*, *SMURF1*, *CKIP1*, and *SIX1* (Maharana and Schlosser, 2018; Park et al., 2013; Piacentino and Bronner, 2018) (Figure 1D). Next, we examined cadherins (*CDH1*, *CDH2*, *CDH6*, *CDH7*, and *CDH11*) involved in NC development and/or expressed in the NPB region (Taneyhill and Schiffmacher, 2017). During induction, mRNA expression of *CDH1* (E-cadherin) and *CDH2* (N-cadherin) are maintained and upregulated on day 4, respectively. *CDH6* and *CDH7*, which are expressed in the NPB of chick and rat embryos (Dady and Duband, 2017; Takahashi and Osumi, 2008), are upregulated and reach peak expression on day 4. Finally, *CDH11*, expressed in chick neural plate (Bell et al., 2004) is also upregulated on day 4, and reaches peak expression on day 6 (Figure 1E).

The embryonic NPB co-expresses specifiers associated with both the neural and non-neural ectoderm. To clarify the identity of neuroepithelial cells, we assessed protein expression of selected factors expressed in the NPB in model organisms, on day 4 of neural induction. We first targeted neural factors PAX6 and ZEB1 (Miyoshi et al., 2006; Thier et al., 2019), and the non-neural factor AP2A (*TFAP2A* [de Crozé et al., 2011]). We also examined SOX1 and TWIST1, which are activated only in post-NPB stages (in the neural tube and during NC EMT, respectively) (Soldatov et al., 2019; Zhang et al., 2010). Flow cytometry analysis revealed partial expression of AP2A (23.37%), PAX6 (19.73%), and ZEB1 (31.63%), while TWIST1 showed reduced number of positive cells (8.58%), and SOX1 expression was largely negative (1.62%) (Figures 1F and 1Fʹ). Since SOX1 is active only on day 6 (Figure S1C), this indicates that day 4 cells express NPB factors and are not fully committed toward either neural or NC fates. Immunostaining of day 4 cells confirmed partial expression of neural (Figure 1G) and non-neural factors (Figure 1Gʹ). Expression of neural factor PAX6 was generally low with few nuclei showing strong expression, as compared with day 6 (Figures 1G and S1B), further suggesting that day 4 cells have not achieved full neural commitment. On day 4, we also observed partial expression and variable nuclear staining intensity for neural factor ZEB1 (Figure 1G) and for non-neural NPB factors AP2A and DLX5 (Figure 1Gʹ) (McLarren et al., 2003). Next, double immunostaining analysis on day 4 showed that expression of neural and non-neural factors overlap to some extent, as a small fraction of the AP2A^+^ nuclei was also positive for ZEB1 and PAX6. In general, double-positive nuclei showed weak staining intensity for at least one of each factor, and strong nuclear co-expression of AP2A and PAX6/ZEB1 was rarely observed (Figures 1H, 1H′, S1D, and S1E).

Together, the above findings show that an NPB-like identity arises on day 4 of the E6 neural induction, which recapitulates embryonic NPB expression patterns. Thus, we renamed this differentiation time point to “NPB-like cells” (NBCs), which were selected to undergo NC induction.

### NBCs Can Be Efficiently Directed toward NBC-Derived NCCs

**Figure 2:**
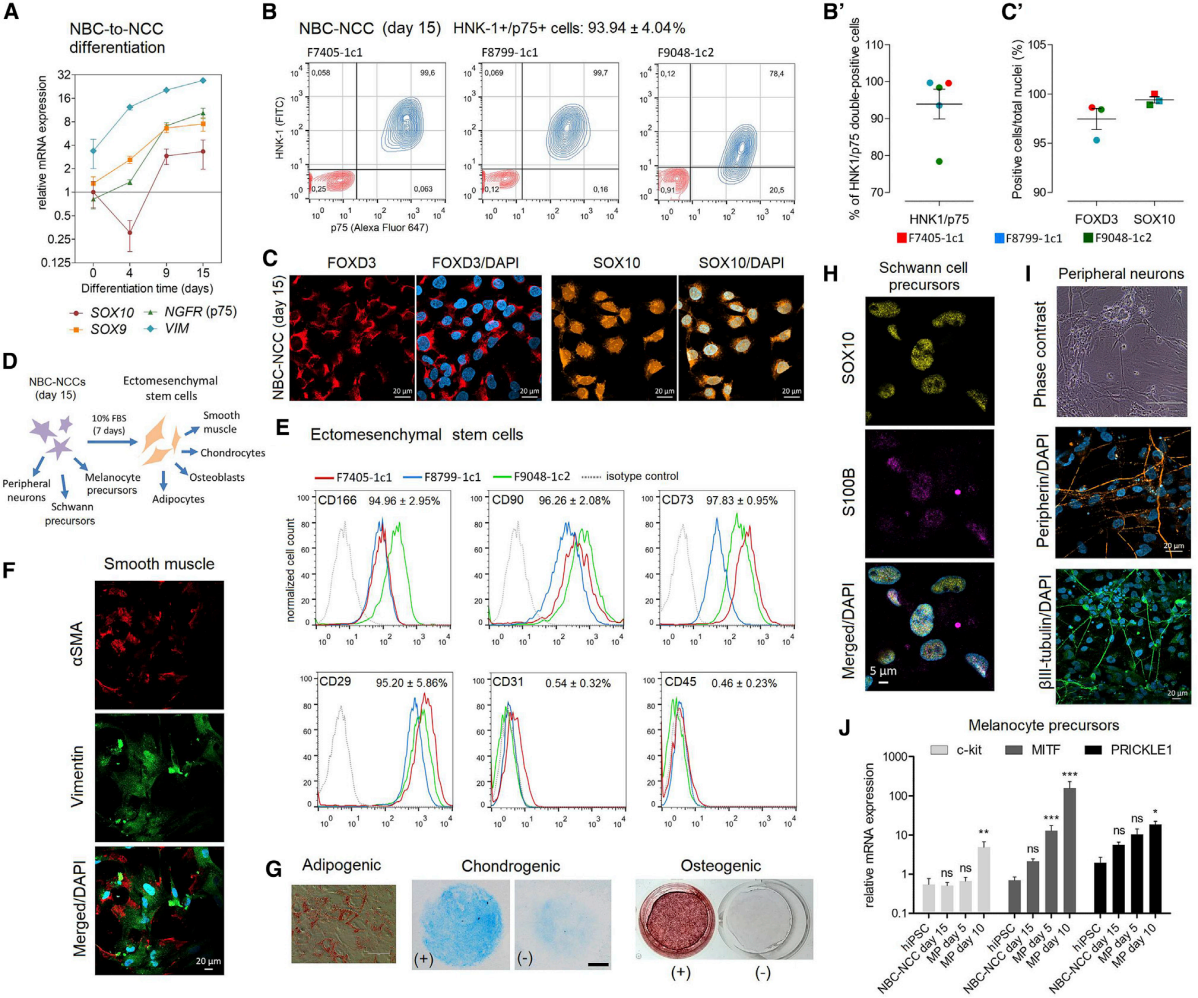
NBCs Can Be Efficiently Converted into Multipotent NCCs (A) qRT-PCR for NC specifiers *SOX9/10*, NC marker *NGFR* (p75), and mesenchymal marker *VIM* (vimentin) during NBC-to-NCC induction. Data are plotted as mean ± SEM (n = 3 biological replicates). (B and B′) Representative flow cytometry analysis for NC markers HNK-1 and p75 depicting double-positive cells (blue) versus isotype (negative) control staining (red). Individual values are plotted in (Bʹ) (n = 3 biological replicates). (C and C′) Representative immunostaining for FOXD3 and SOX10; quantification of positive cells/total nuclei is plotted in (Cʹ). Bars represent means ± SEM (n = 3 biological replicates). (D) Diagram depicting terminal differentiations of NBC-NCCs. (E) Flow cytometry profiles of ectomesenchymal stem cells differentiated from NBC-NCCs for mesenchymal markers CD73, CD90, CD166, and CD29, endothelial marker CD31, and hematopoietic marker CD45. Quantification was done in relation to isotype (negative) controls (n = 3 biological replicates). (F–I) Representative differentiations of NBC-NCCs (n = 3 biological replicates): (F) αSMA/Vimentin staining (smooth muscle cells). Scale bar, 20 μm. (G) Oil red O, alizarin red, and Alcian blue staining (adipogenic, osteogenic, and chondrogenic differentiation, respectively). Scale bars, 400 μm (oil red O) and 1 mm (Alcian blue). +, differentiated cells; −, negative control. (H) SOX10/S100B staining (glial/Schwann cell precursors). Scale bar, 5 μm. (I) Morphology of peripheral neurons and βIII-tubulin and peripherin staining. Scale bar, 20 μm. (J) Melanocyte differentiation. qRT-PCR for melanocyte precursor (MP) markers *MITF*, *c-kit*, and *PRICKLE1*. Means ± SEM were plotted against undifferentiated hiPSCs (n = 3 biological replicates). ^∗∗∗^p < 0.001, ^∗∗^p < 0.01, ^∗^p < 0.05; one-way ANOVA with Dunnett's correction.

NCCs were induced from NBCs with a modified method that uses the same minimal E6 medium base as NBCs, supplemented with basic fibroblast growth factor (bFGF), an Activin pathway blocker (SB431542), and a WNT pathway activator (CHIR99021), for 15 days (Miller et al., 2017). During NBC-to-NCC induction, we observed gradual upregulation of NC specifiers *SOX9* and *SOX10*, in addition to NC markers *NGFR* (p75) and *VIM* (Vimentin) (Figure 2A). Flow cytometry analysis on day 15 demonstrated 93.94% of double-positive cells for NC markers p75/HNK-1 (Figures 2B and 2Bʹ), while immunofluorescence quantification revealed 97.46% of FOXD3^+^ and 99.41% SOX10^+^ cells (Figures 2C and 2Cʹ).

We further characterized day-15 NBC-derived NCCs (NBC-NCCs) via differentiation into NC derivatives. NBC-NCCs were first converted to ectomesenchymal stem cells (Figure 2D), which showed positive staining (>94%) for mesenchymal markers CD29, CD73, CD166, and CD90, and negative staining (<0.45%) for endothelial marker CD31 and hematopoietic marker CD45 (Figure 2E). These cells were further differentiated toward αSMA^+^/Vimentin^+^ smooth muscle (Figure 2F), and adipocyte, osteoblast, and chondrocyte lineages, as demonstrated by oil red O, alizarin red, and Alcian blue staining, respectively (Figure 2G). Day 15 NBC-NCCs were also directed toward SOX10^+^/S100B^+^ Schwann cell precursors (Figure 2H), in addition to peripheral neurons expressing βIII-tubulin and peripherin (Figure 2I). Finally, differentiation into melanocyte precursors showed progressive upregulation of melanocyte markers *c-kit*, *MITF*, and *PRICKLE1* (Hosaka et al., 2019) (Figure 2J). These results confirm the NC identity of NBC-NCCs and show efficient production of NCCs from NBCs.

### NBC-to-NCC Induction Recapitulates NC Specification and EMT

**Figure 3:**
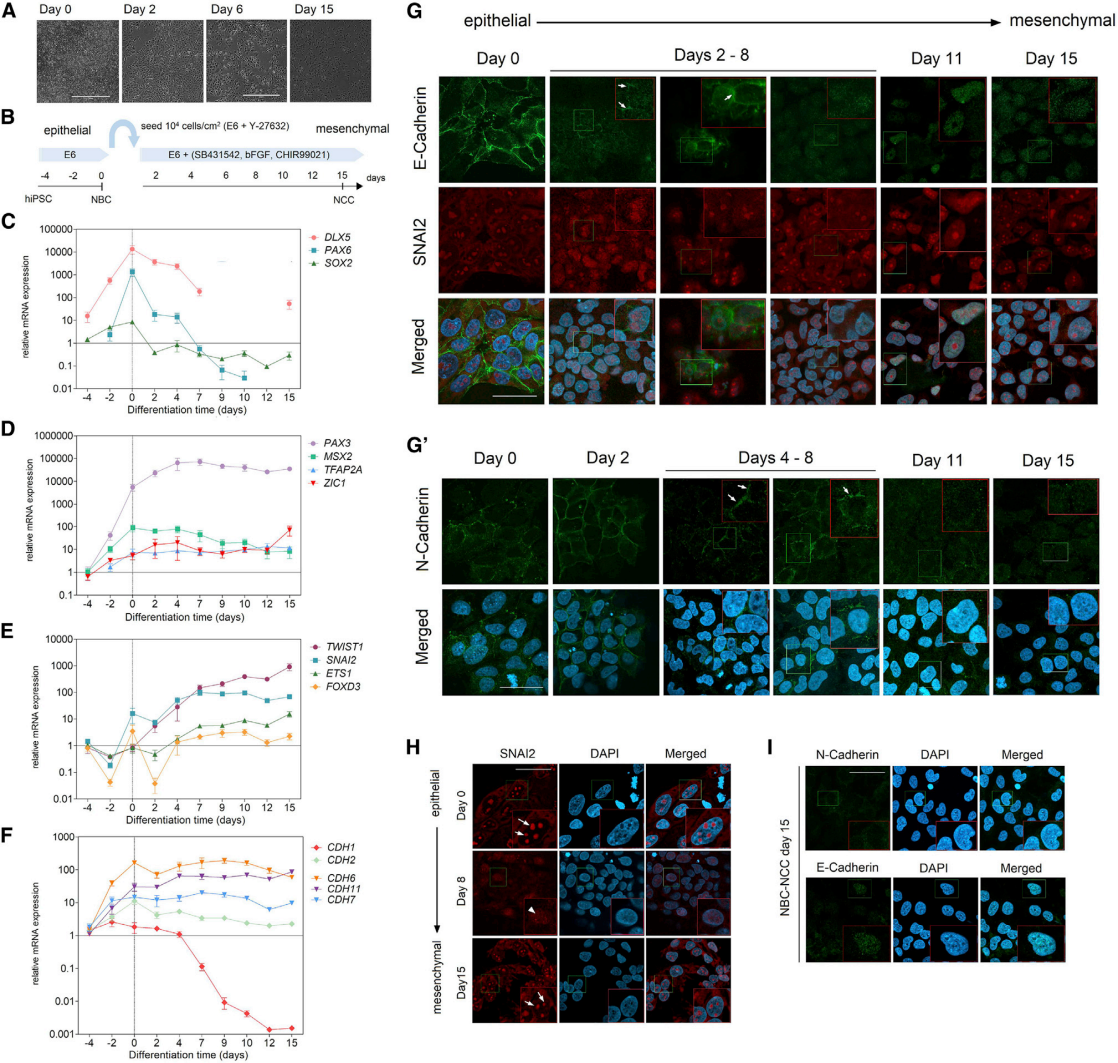
NBC-to-NCC Differentiation Recapitulates NC Specification and EMT (A) Representative phase-contrast micrographs depicting epithelial-to-mesenchymal morphological changes during NBC-to-NCC differentiation. Scale bars, 400 μm. (B) Schematic representation of the differentiation setup encompassing hiPSC-to-NBC followed by NBC-to-NCC derivation. (C–F) Time series qRT-PCR analysis of (C) NPB factors *PAX6*, *SOX2*, and *DLX5*, (D) NPB/NC specifiers *PAX3*, *MSX2*, *TFAP2A*, and *ZIC1*, (E) NC specifiers/EMT inducers *TWIST1*, *SNAI2*, *ETS1*, and *FOXD3*, and (F) cadherins involved in NC specification/EMT *CDH1*, *CDH2*, *CDH6*, *CDH7*, and *CDH11*. Expression values were plotted relative to day −4 except for *PAX6* and *TFAP2A* (day −2). qRT-PCR data are plotted as mean ± SEM (n = 3 biological replicates). (G and G′) Time series immunofluorescence analysis showing expression patterns for (G) E-cadherin (CDH1) and SNAI2, and for (G′) N-cadherin. Arrows indicate E/N-cadherin expression at cell-cell junctions. Panels for F8799-1c1 are shown here as representative and in Figures S2 and S3 (n = 3 biological replicates). (H) Representative immunofluorescence showing SNAI2 expression patterns observed in: NBCs (day 0), during NBC-NCC induction (observed from days 2 to 8), and in NBC-NCCs (day 15). n = 3 biological replicates. (I) Representative immunostaining showing mesenchymal expression patterns for E-cadherin and N-cadherin in NBC-NCCs on day 15 (n = 3 biological replicates). For (G)–(I) Scale bars, 45 μm. Insets: 2× magnification. Related to Figures S2 and S3.

From day 0 to 15 of NBC-to-NCC induction, we observed gradual morphological changes reminiscent of EMT in all samples. On day 2, cells were still organized as an epithelial monolayer with evident cell-cell adhesions, which were gradually lost until day 15 (Figure 3A). To confirm whether these changes result from activation of the NC specification/EMT program, we performed time series analysis of mRNA expression for several specifiers and markers. To better visualize expression dynamics associated with early NC development, we included the initial 4 days (hiPSC-to-NBC) followed by the 15 days (NBC-to-NCC) of induction, where NBCs correspond to day 0, and hiPSCs to day −4 (Figure 3B).

After upregulation from hiPSCs to NBCs, neural/NPB factors *PAX6* and *SOX2* were downregulated during NBC-NCC induction, with undetectable *PAX6* expression by the end (days 12 and 15); after upregulation on day 0 (NBCs), NPB factor *DLX5* was also downregulated, remaining active until day 7, and was later reactivated on day 15, possibly reflecting later NCC functions in pharyngeal arch patterning (Shimizu et al., 2018) (Figure 3C). After initial upregulation in NBCs, expression of NPB factors also required in NC specification was either maintained (*TFAP2A*/*MSX2/ZIC1*) or further increased (*PAX3*) until day 15 (Figure 3D). From days 0 to 15, we also observed upregulation of NC specifiers/EMT inducers *TWIST1*, *SNAI2*, *FOXD3*, and *ETS1* (Figure 3E). Finally, expression of epithelial cadherins *CDH1/2* was already observed on day 0 and then reduced, with *CDH1* showing pronounced downmodulation from day 7 onward; conversely, mesenchymal cadherins *CDH6/7/11* either retained high expression (*CDH6*/*7*) or were further upregulated (*CDH11*) from day 0 to 15 (Figure 3F). These observations show that the differentiating NBC-NCC populations recapitulate the transcriptional program for NC specification/EMT.

During EMT, de-epithelization occurs via deconstruction of cell-cell junctions by mechanisms that include silencing of adhesion molecules by SNAI1/2 and cleavage of epithelial cadherins at the plasma membrane (Lamouille et al., 2014). We investigated protein expression/localization of CDH1 (E-cadherin) and its transcriptional repressor SNAI2 during NBC-to-NCC induction. Immunostaining demonstrated E-cadherin localized to cell-cell contact sites from day 0 to 2 in all samples (Figures 3G and S2). E-Cadherin was detected at cell boundaries until day 2 for F9048-1c2 (Figure S2A), day 4 for F8799-1c1 (Figure S2B), and day 8 for F7405-1c1 (Figure S2C). On average, E-cadherin disappearance from these locations takes place from days 2 to 8; afterward, E-cadherin was no longer expressed in plasma membranes (Figure 3G). SNAI2 protein expression was also confirmed on day 0 (NBCs), localized to DAPI^−^ nuclear foci. During induction, this occasionally shifted to a scattered nuclear SNAI2 staining pattern (e.g., day 2 of F8799-1c1, day 8 of F7405-1c1, and F9048-1c2). Nevertheless, nuclear expression of SNAI2 could be observed in all time points, being strongly expressed in nuclear foci at the end of induction (Figures 3H and S2A–S2C).

Like E-cadherin, N-cadherin (CDH2) is lost at intercellular contact sites as cells acquire mesenchymal features. Initially, N-cadherin was observed at those sites from days 0 to 4 in all samples (Figures 3Gʹ and S3). From day 4 onward, N-cadherin could still be detected in membrane patches between neighboring cells until day 6 in F7405-1c1 (Figure S3A), and day 8 in F8799-1c1 and F9048-1c2 (Figures S3B and S3C). In one sample (F7405-1c1), diffuse N-cadherin expression could still be observed on day 11 in some plasma membranes, although cells were already fully individualized on this day (Figure S3C). On average, strong expression and localization of N-cadherin between cells become less evident from days 4 to 8 (Figure 3Gʹ). On day 15, the mesenchymal staining pattern of N-cadherin appeared to be cytoplasmic, while E-cadherin showed evident nuclear accumulation in all samples (Figure 3I). Overall, we observed gradual disappearance of E/N-cadherin at epithelial cell-cell junctions between days 2 and 8, which roughly coincides with the upregulation time frame of NC specifiers/EMT inducers *TWIST1*, *SNAI2*, *ETS1*, and *FOXD3* (Figure 3E).

### Cells Undergoing EMT Are Distinguished by TWIST1 Upregulation on NBC-NCC Day 7

**Figure 4:**
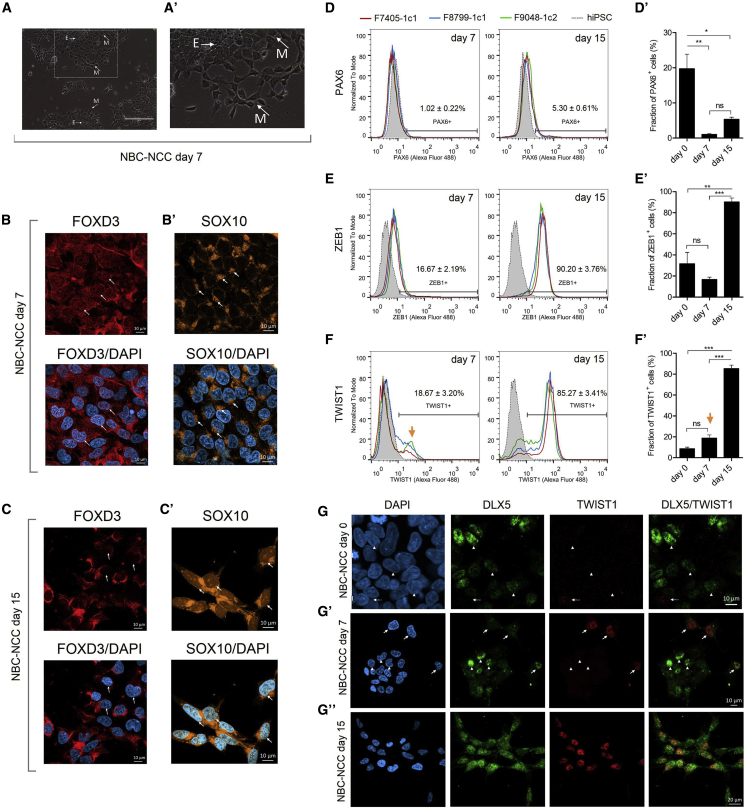
EMT Cell-State Transitions on Day 7 of NBC-NCC Induction (A and A′) Representative phase-contrast micrographs depicting morphology of day 7 NBC-NCCs; higher magnification is shown in (Aʹ). E, epithelial-like cell clusters; M, mesenchymal-like cells. Scale bar, 200 μm. (B–C′) Representative immunostaining FOXD3 and SOX10 on days 7 (B and B′) and 15 (C and C′) (n = 3 biological replicates). Arrows in (B and C) indicate nuclear expression and faint nuclear expression of FOXD3, respectively. Arrows in (B′ and C′) show perinuclear aggregates and nuclear expression of SOX 10, respectively. Scale bar, 10 μm. (D–F) Flow cytometry analysis on days 7 and 15 of induction for PAX6 (D), ZEB1 (E), and TWIST1 (F). (D′–F′) Flow cytometry results from days 7 and 15 were plotted in comparison with day 0. Data are plotted as mean ± SEM (n = 3 biological replicates); yellow arrows indicate a TWIST1^+^ subpopulation. ^∗∗∗^p < 0.001, ^∗∗^p < 0.01, ^∗^p < 0.05; ns, not significant; one-way ANOVA with Dunnet's correction. (G–G″) Representative double immunostaining analysis for DLX5xTWIST1 in NBC-NCCs on day 0; arrowheads point to DLX5^+^ cells; dashed arrows point to TWIST1^+^ cells. (G′ and G″) Representative double immunostaining for DLX5xTWIST1 on day 7 (G′), and day 15 (G″) of NBC-NCC induction (n = 3 biological replicates). Arrowheads point to DLX5^+^ cells; arrows point to DLX5^+^/TWIST1^+^ cells. Scale bars, 10 μm (days 0/7) and 20 μm (day 15). Related to Figure S4.

Despite the variations in cadherin dynamics between days 2 and 8, we observed that NBC-NCCs were, around day 7, invariably composed of several dense, epithelial-like cell clusters surrounded by individualized, mesenchymal-like cells (Figures 4A and 4A′). Therefore, we focused on this day to search for additional molecular signatures compatible with NC EMT. NC specifiers FOXD3 and SOX10 are also implicated in EMT (Fairchild et al., 2014; McKeown et al., 2005), so we first investigated these factors to confirm acquisition of NC identity and induction of EMT on day 7 of NBC-NCC differentiation. We observed nuclear and cytoplasmic FOXD3 expression (Figure 4B), while SOX10 expression was more cytoplasmic and localized to perinuclear aggregates (Figure 4Bʹ). After completion of NC induction, day 15 NBC-NCCs show less pronounced nuclear expression of FOXD3 (Figures 4C and 2C), and show SOX10 expression in the nuclei and cytoplasm (Figures 4Cʹ and 2C). These results confirm that cells are already undergoing NC specification/EMT by day 7, and suggest that nuclear functions of FOXD3 precede those of SOX10 during this process. This further shows that NBC-NCC induction recapitulates temporal expression patterns observed in embryonic NC development, in which FOXD3 acts in early NC specification, preceding SOX10 functions in premigratory NCCs (Simões-Costa et al., 2012).

Next, flow cytometry analysis during NBC-NCC induction confirmed downregulation of PAX6, which was mostly negative on day 7 (1.02%) and day 15 (5.30%) (Figures 4D and 4D′), while EMT inducers ZEB1 and TWIST1 were upregulated: in comparison with day 0, day 7 cells maintained partial ZEB1 expression (16.67%), which rose to 90.20% on day 15 (Figures 4E and 4E′), while TWIST1 was expressed in 18.67% of day 7 cells, increasing to 85.27% on day 15. Importantly, assessment of TWIST1 expression on day 7 clearly distinguishes a positive population diverging from TWIST1^−^ cells, albeit not reaching statistical significance compared with day 0 (Figures 4F and 4F′).

Mouse NCCs express DLX5 around delamination/EMT (Soldatov et al., 2019), and our results showed that NBC-NCCs express this factor until day 7 of NBC-NCC induction, after which it is temporarily inactivated (Figure 3C). Considering that TWIST1^+^ cells appear on this day, we targeted DLX5 and TWIST1 with double immunostaining on days 0, 7, and 15 to further characterize EMT-associated cell-state changes. On day 0, we observed some TWIST1 expression, which largely did not overlap with DLX5 (Figures 4G and S4C). On day 7, this pattern shifted to several DLX5^+^/TWIST1^−^ cells accompanied by double-positive cells, suggesting that TWIST1^+^ cells derive from DLX5^+^ cells undergoing EMT (Figures 4Gʹ and S4Cʹ). Furthermore, DLX5^+^/TWIST1^+^ cells were also detected on day 15 (Figures 4G″ and S4C″), confirming re-activation of *DLX5* on this day (Figure 3C). Together, these observations further corroborate recapitulation of EMT in this model and show that an emergent subpopulation distinguished by activation of TWIST1 can be detected within day 7 NBC-NCCs.

## Discussion

Here, we report an improved approach to generate NCCs from an NPB-like intermediate stage that enables investigation of early NC formation in humans. This was achieved by coupling independent strategies for neural and NC induction (Lippmann et al., 2014; Miller et al., 2017). Although these methods are not essentially new, we show that their joint usage enables stepwise recapitulation of NC specification and EMT that initiate within the embryonic NPB, paving new ways to examine these early steps of NC development under a simple experimental framework.

We report that neural induction with the E6 method confers an NPB-like state to differentiating hiPSCs in 4 days. The original work describing this method focused on derivation of neural-committed cells in 6 days without blocking the BMP pathway, and NPB identity was not assessed (Lippmann et al., 2014). *In vivo* work has shown that the NPB domain is specified under intermediate BMP activity, allowing partial expression of neural and non-neural specifiers that are co-expressed in some cells (Roellig et al., 2017). We show that cells on day 4 of the E6 induction (NBCs) recapitulate expression patterns for several NPB specifiers and markers, and show partial expression of neural and non-neural NPB factors (AP2A, DLX5, PAX6, and ZEB1) with some degree of co-expression, at least for AP2A and PAX6/ZEB1. Thus, we propose that lack of BMP blockade could be setting permissive culture conditions in which intermediate (native) BMP activity allows acquisition of an NPB-like identity on day 4 of induction, which has not been reported for the E6 method. We acknowledge that the markers evaluated here are not exclusive to the NPB, so further studies are necessary to shed light on human NPB specification and to accurately determine the proportion of bona fide NPB cells within NBCs. Nevertheless, given that most protocols to derive NCCs do not include an NPB stage, NBCs could be an interesting approach to clarify the requirements in cell fate decisions leading to NC development within the NPB territory.

We show that redirecting NBCs toward the NC fate generates NCCs with high efficiency, resulting in 93.94% of HNK1^+^/p75^+^ cells and >97% SOX10^+^ and FOXD3^+^ cells, on average. Although several strategies to produce human NCCs have been devised, conversion rates vary across different works, and may range from 15% to 90% HNK1^+^/p75^+^ and 30%–80% of SOX10^+^ NCCs (Hackland et al., 2017; Lee et al., 2010; Leung et al., 2016; Menendez et al., 2013; Tchieu et al., 2017). The high efficiency of NBC-to-NCC conversion here reported could be attributed to the presence of the NBC stage, which may prime cells in a pre-NC (i.e., NPB) state that facilitates acquisition of the NC fate, as this more closely abides by the developmental steps required for NC specification *in vivo*, compared with direct NCC conversion from hiPSCs. In addition, in this work, we used minimal essential medium in all culture steps, which may be positively influencing NCC yield, since non-essential medium formulations may contain additives (e.g., bovine serum albumin [BSA]) that affect endogenous BMP signaling and result in variable NCC conversion rates (Hackland et al., 2017).

To date, few studies have explored the dynamic changes in cellular properties that take place at different stages after NC induction. In humans, the study of EMT is largely restricted to cancer cells and fibrosis, and knowledge on human NC EMT has been inferred from observations in animal models (Theveneau and Mayor, 2012; Yang et al., 2020). Here, we provide time series characterization of the expression patterns of several specifiers and EMT markers ascertained in these models to show that NBC-to-NCC induction recapitulates molecular events of NC specification and EMT. From day 0 to 15 of NBC-to-NCC induction, we observed downregulation of some NPB factors (*DLX5*, *PAX6*, and *SOX2*), increment or maintenance in expression of factors involved both in NPB and NC specification (*PAX3*, *MSX2*, *TFAP2A*, and *ZIC1*), and upregulation of NC specifiers/EMT inducers (*SNAI2*, *TWIST1*, *ETS1*, *FOXD3*, and ZEB1). These changes were accompanied by downregulation of *CDH1/2* in favor of mesenchymal cadherins *CDH6/7/11*, a hallmark of NC EMT (Strobl-Mazzulla and Bronner, 2012). *In vivo*, NC EMT depends on proper regulation of multiple cadherins by EMT inducers, which was demonstrated here by gradual morphological and molecular hallmarks of EMT in the course of 15 days, including upregulation of EMT inducers *TWIST1*, *SNAI2*, *ETS1*, and *FOXD3* coupled to downregulation of E/N-cadherin mRNA, and reduction of E/N-cadherin protein localization to cell-cell contact sites from days 2 to 8. Despite the variation in timing for these events across samples, EMT-related changes in gene expression and cadherin localization take place between days 2 and 8. In line with this, NBC-NCCs on day 7 show protein expression patterns specific for NCCs undergoing EMT, including expression of EMT inducer ZEB1, upregulation of EMT inducer TWIST1, and downregulation of NPB factor PAX6. Moreover, this time point permits detection of specific EMT cell fate transition events, illustrated here by acquisition of TWIST1 by a subpopulation of cells positive for DLX5, which is expressed in the delamination/EMT phase of mouse NC development. Of interest, TWIST1 confers ectomesenchymal bias to cranial NCCs upon EMT (Soldatov et al., 2019), and DLX5 is activated after EMT in cranial NCCs to regulate patterning of the pharyngeal arches (Shimizu et al., 2018). Our results show that over 85% of NBC-NCCs express TWIST1 at the end of NC induction, which, at least in some of them, is co-expressed with DLX5. This suggests that NBC-NCC induction favors production of cranial NCCs with ectomesenchymal bias, and points to potential applications in modeling alterations related to pharyngeal arch patterning, such as the mandibular defects of auriculo-condylar syndrome (Clouthier et al., 2013).

After the shift from epithelial-to-mesenchymal cell states, E-cadherin expression is localized to cell nuclei instead of being completely ablated. These observations are in line with the novel roles ascribed to cadherins in NC EMT and migration in animal models, in which cadherin fragments can be translocated to nuclei to activate specific expression programs (Taneyhill and Schiffmacher, 2017). Our results support the notion that cadherins exert functions beyond adhesion, and indicate that E-cadherin may play novel roles in human NC development. Further investigation is required to shed light on the roles played by cadherins and other factors involved in human NC specification/EMT, as well as to elucidate the similarities and dissimilarities between humans and animal models.

Given the lack of human *in vitro* models of NC EMT, the NBC-NCC strategy could be applied to investigate both normal human development and disorders associated with this process. These include, besides congenital disorders, NC-derived tumors, such as neuroblastoma, melanoma and others, in which the EMT program is recapitulated to drive malignancy and metastasis (Maguire et al., 2015). In addition, the simple and efficient method for generating NBC-NCCs reported here could be further developed toward drug/genetic screening and cytotoxicity tests. Finally, it provides means to investigate the functional effects of genetic variants detected by next-generation sequencing or other methods, which is a current challenge in Medical Genetics research.

## Experimental Procedures

### Ethics Statement

The Ethics Committee of Instituto de Biociências at Universidade de São Paulo, Brazil (1.463.852) approved the experimental procedures. Human subjects donated biological samples after providing signed informed consent.

### Sample Setup

Three hiPSC lines derived from healthy individuals (F7405-1c1, F8799-1c1, and F9048-1c2) were used in all experiments, except for immunostaining analysis of day 7 NBC-NCCs (Figures 4 and S4), in which F8799-1c1 was replaced by one additional sample (F7007-1c2). This sample replicates findings, such as expected morphology for NBC-NCC induction and expression of NCC markers SOX10 and FOXD3 (Figures S4A and S4B), so we opted to include it in this specific assay.

All experiments were performed with at least n = 3 biological replicates.

### Generation and Maintenance of hiPSCs

Three hiPSC lines were reprogrammed from dermal fibroblasts (F9048-1c2, F7405-1c1, and F7007-1c2) and one from erythroblasts (F8799-1c1) according to previous protocols (Miller et al., 2017). hiPSCs were maintained in E8 medium (Thermo Fisher Scientific) supplemented with Normocin (Invivogen). Cells were cultured in Matrigel (BD Biosciences)-coated vessels, and passaged with Accutase (Thermo Fisher Scientific). These hiPSC lines had already been characterized as reported elsewhere (Ishiy et al., 2015; Miller et al., 2017).

### Differentiation of hiPSCs to NBCs

hiPSCs were conditioned to E8 medium for at least two passages before differentiation toward NBCs via the E6 method (Lippmann et al., 2014). In brief, hiPSCs were seeded onto 60-mm Matrigel-coated dishes (1 × 10^5^ cells/cm^2^) in E8 medium with Normocin and 5 μM Y-27632, and 1 day post-seeding, medium was changed to E6 medium (Thermo Fisher Scientific) with Normocin and replenished daily for 6 days.

### Differentiation of NBCs to NCCs

NBCs were passaged with Accutase, and seeded in Matrigel-coated dishes (3 × 10^4^ cells/cm^2^) in E6 with 5 μM Y-27632. In the following day, medium was changed to NCC medium (Miller et al., 2017), composed of E6 supplemented with Normocin, 8 ng/mL bFGF (Thermo Fisher Scientific), 20 μM SB431542 (Tocris), 1 μM CHIR99021 (Sigma). Cells were split before reaching confluence with Accutase at room temperature (RT) every 2–3 days. Since cells continue to replicate as differentiation takes place, harvesting was timed with subculturing to comprise the 15 days of differentiation in eight time points for qRT-PCR and immunofluorescence of E/N-cadherin and SNAI2.

### Differentiation of NBC-NCCs to Ectomesenchymal Derivatives

NCC-derived ectomesenchymal stem cells were induced with mesenchymal medium consisting of DMEM/F12 supplemented with 10% fetal bovine serum (FBS), 2 mM GlutaMAX, 0.1 mM non-essential amino acids (Thermo Fisher Scientific), and Normocin. NCCs were seeded at 2 × 10^4^ cells/cm^2^ onto non-coated 60-mm dishes, differentiated for 6 days, and passaged with TrypLE Express (Thermo Fisher Scientific). Medium was changed every 3 days.

To produce smooth muscle, cells were treated with DMEM/F12 supplemented with 5% FBS and 10 ng/mL transforming growth factor β1 (PeproTech) for 3 weeks (Wang et al., 2012) with medium changes every 3 days. Osteogenic, chondrogenic, and adipogenic differentiation were induced with commercial kits (StemPro Osteogenesis, Chondrogenesis, and Adipogenesis kits, Thermo Fisher Scientific) following the manufacturer's recommendations. After 21 days of differentiation, osteoblasts and adipocytes were stained with alizarin red-S, and oil red O as reported previously (Miller et al., 2017). Chondrocytes were induced for 7 days in micromass cultures; in brief, cells were resuspended at 10^4^ cells/μL in mesenchymal medium, and 5-μL droplets were deposited in 24-well plates and incubated at 37°C in 5% CO_2_. After 45 min, 400 μL of chondrogenesis medium was carefully added to each well. Alcian blue staining was performed according to the manufacturer's instructions. Undifferentiated ectomesenchymal stem cells were used as negative controls.

### Differentiation of NBC-NCCs to Peripheral Neurons, Schwann, and Melanocyte Precursors

For Schwann precursors, NBC-NCCs were seeded at 5 × 10^4^ cells/cm^2^ on Matrigel-coated dishes and cultured for 40 days in NC medium supplemented with 20 ng/mL heregulin-β1 (PeproTech) (Liu et al., 2012); medium was changed every 3 days. To generate peripheral neurons, NBC-NCCs were seeded at 10^5^ cells/cm^2^ on Matrigel-coated dishes, and were differentiated for 10 days in DMEM/F12 supplemented with 1% penicillin-streptomycin solution, 1× N2 supplement (Thermo Fisher Scientific), 10 ng/mL brain-derived neurotrophic factor, 10 ng/mL glial cell line-derived neurotrophic factor, 10 ng/mL NT-3 (PeproTech), 10 ng/mL NGF (BioLegend), 0.5 mM db-cAMP, and 200 μM ascorbic acid (Menendez et al., 2013). Melanocyte precursors were induced by seeding 5 × 10^4^ cells on Matrigel-coated dishes in E6 medium supplemented with Normocin, 1 μM CHIR99021, 100 nM endothelin-3 (Tocris), and 1 μM SJ000291942 (Sigma). Medium was changed every 3 days.

### Real-Time Quantitative PCR

One microgram of RNA was obtained with a NucleoSpin RNA II kit (Macherey-Nagel) and converted into cDNA with Superscript IV (Thermo Fisher Scientific) according to the manufacturer's recommendations. Reactions were performed with 2× Fast SYBR Green PCR Master Mix (Thermo Fisher Scientific) and 50–400 nM of each primer (Table S1). Fluorescence was detected in a 7500 Fast Real-Time PCR System or QuantStudio 5 under standard cycling conditions. Amplification efficiencies were determined by serial cDNA dilutions or calculated with LinRegPCR (Ramakers et al., 2003). NormFinder (Andersen et al., 2004) was used to determine the most stable endogenous control (among *ACTB*, *TBP*, *HMBS*, *GAPDH*, and *HPRT1*). Relative expression was calculated as elsewhere (Pfaffl, 2001).

### Immunofluorescence Analyses

Cells were fixed in 4% paraformaldehyde for 20 min at RT and permeabilized with PBS 0.2% Triton X-100 for 30 min followed by blocking with PBS 5% BSA for 1 h at RT. Cells were incubated with primary antibodies overnight at 4°C under gentle agitation, washed 3× with PBS, and incubated with secondary antibodies at 4°C for 1 h. Cells were counterstaining with DAPI for 2 min at RT. Antibodies used in this study are detailed in Table S2. Slides were analyzed under a confocal microscope (Zeiss LSM 800) and captured z stacks were processed on ImageJ or ZEN Blue. To assess induction efficiency of NBC-NCCs on day 15, FOXD3- and SOX10-positive cells were counted in relation to total nuclei in four different fields. At least 120 nuclei in total were counted for each sample.

### Flow Cytometry Analyses

To assess expression of NPB and EMT markers, cells were dissociated with Accutase, washed once with PBS, and fixation, permeabilization, and antibody incubation were performed with FIX & PERM (Thermo Fisher Scientific). Cells were incubated for 1 h with primary antibodies at 4°C, and 1 h with secondary antibodies at 4°C (Table S2). To account for non-specific staining, quantification of positive cells was carried out in relation to hiPSCs, which should be negative for PAX6, AP2A, ZEB1, and TWIST1. For SOX1, quantification was done in relation to NBCs incubated with secondary antibody only, as staining specificity was confirmed in day 6 cells compared with day 4 (Figures 1F and S1C).

Day 15 NBC-NCCs were double stained for p75/HNK-1 with conjugated antibodies. Ectomesenchymal stem cells were stained with antibodies for CD29, CD31, CD45, CD90, CD73, and CD166. Expression was quantified in relation to appropriate immunoglobulin-matching isotype controls to calibrate for non-specific staining (Table S2).

At least 5,000 events were acquired in a FACSAria II equipment and analyzed on FlowJo.

### Statistical Analyses

Gene expression values were log-transformed before statistical analyses. One-way ANOVA was conducted to compare the effect of differentiation (days) on gene expression across samples. When means were significantly different (p < 0.05), Dunnett's multiple comparison test was performed, and significance was indicated in comparison with day 0 of differentiation, unless otherwise stated. Analyses were carried out on GraphPad Prism.

## Author Contributions

G.S.K. and C.M.M. conceptualized the work and designed the experiments. G.S.K., C.M.M., D.d.P.M., G.P.-G., G.S.P.H., L.C.C.-J., E.G., and M.R.P.-B. contributed with data acquisition, analysis and/or interpretation. G.S.K., C.M.M., and M.R.P.-B. wrote the manuscript.

## Supplemental Information

Document S1. Figures S1–S4 and Tables S1 and S2

Document S2. Article plus Supplemental Information
