# Supplementary material for: Recapitulation of Neural Crest Specification and EMT via Induction from Neural Plate Border-like Cells
Source: Stem Cell Reports. 2020 Aug 27;15(3):776–88. doi: 10.1016/j.stemcr.2020.07.023 (PMC7486307; doi:10.1016/j.stemcr.2020.07.023)
Supplement: Document S1. Figures S1–S4 and Tables S1 and S2 [file mmc1.pdf]

**Stem Cell Reports, Volume *15***

## Supplemental Information

# Recapitulation of Neural Crest Specification and EMT via Induction from Neural Plate Border-like Cells

**Gerson Shigeru Kobayashi, Camila Manso Musso, Danielle de Paula Moreira, Giovanna Pontillo-Guimarães, Gabriella Shih Ping Hsia, Luiz Carlos Caires-Júnior, Ernesto Goulart, and Maria Rita Passos-Bueno**

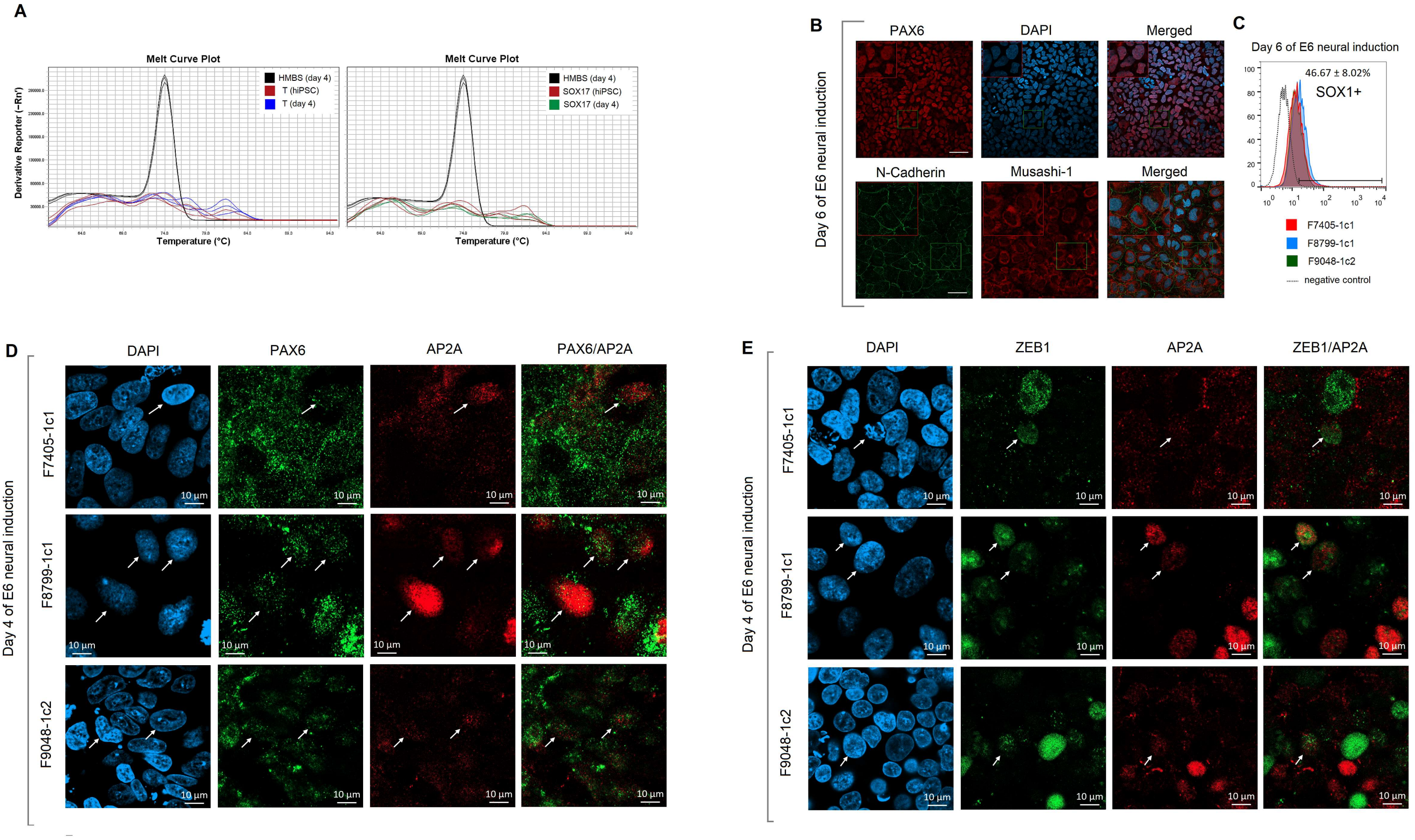

A
Melt Curve Plot
HMBS (day 4)
T (hiPSC)
T (day 4)
Derivative Reporter (-Rn')
Temperature (°C)
Melt Curve Plot
HMBS (day 4)
SOX17 (hiPSC)
SOX17 (day 4)
Temperature (°C)
B
Day 6 of E6 neural induction
PAX6
DAPI
Merged
N-Cadherin
Musashi-1
Merged
C
Day 6 of E6 neural induction
46.67 ± 8.02%
SOX1+
F7405-1c1
F8799-1c1
F9048-1c2
negative control
D
Day 4 of E6 neural induction
DAPI
PAX6
AP2A
PAX6/AP2A
F7405-1c1
F8799-1c1
F9048-1c2
10 µm
E
Day 4 of E6 neural induction
DAPI
ZEB1
AP2A
ZEB1/AP2A
F7405-1c1
F8799-1c1
F9048-1c2
10 µm

**Figure S1 (Related to Figure 1): A)** Dissociation curves of PCR products generated with RT-qPCR for mesodermal specifier *T* and endodermal specifier *SOX17* on days 0 (hiPSCs) and 4 of neural induction. Housekeeping control *HMBS* is shown for comparison. n=3 biological replicates. **B)** Representative immunostaining for neural markers PAX6 and Musashi-1/N-cadherin on day 6 of neural induction. Insets: 2x magnification. Scale bar: 45 µm. **C)** Flow cytometry analysis of SOX1 on day 6 of neural induction. Positively stained cells were quantified in relation to secondary antibody as negative control (see Experimental Procedures – Flow Cytometry analyses); n=3 biological replicates. **D, E)** Double immunostaining analysis for AP2AxPAX6 **(D),** and AP2AxZEB1 **(E)**. White arrows point to double-positive nuclei. Scale bars: 10 µm.

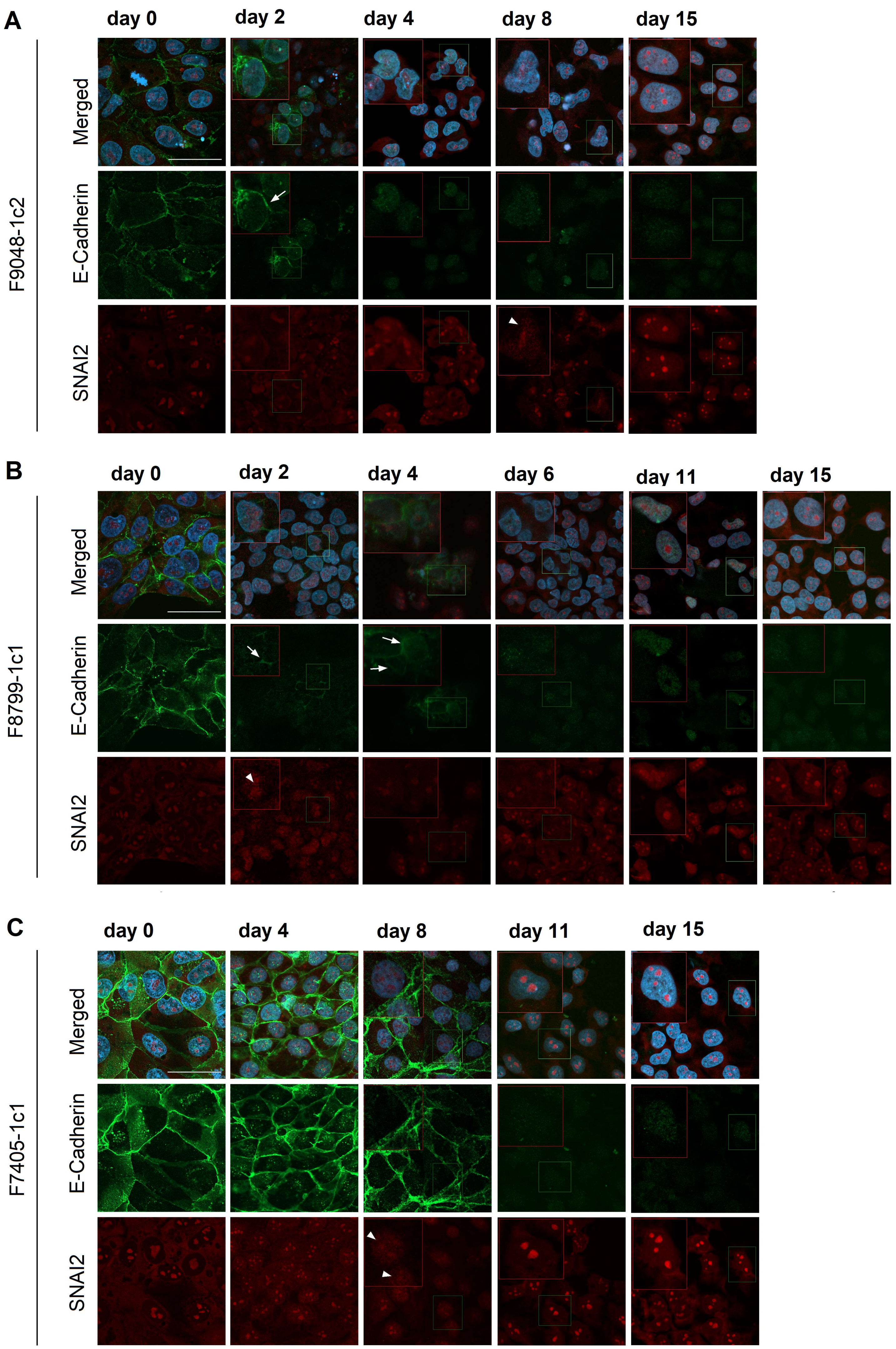

A
day 0
day 2
day 4
day 8
day 15
F9048-1c2
Merged
E-Cadherin
SNAI2
B
day 0
day 2
day 4
day 6
day 11
day 15
F8799-1c1
Merged
E-Cadherin
SNAI2
C
day 0
day 4
day 8
day 11
day 15
F7405-1c1
Merged
E-Cadherin
SNAI2

**Figure S2 (Related to Figure 3):** Time-series immunofluorescence analysis showing expression patterns for E-cadherin (CDH1) and SNAI2 during NBC-to-NCC differentiation in all samples. Arrows indicate E-cadherin expression at cell-cell junctions. Arrowheads indicate scattered nuclear SNAI2 expression. Scale bars: 45 µm. Insets: 2x magnification.

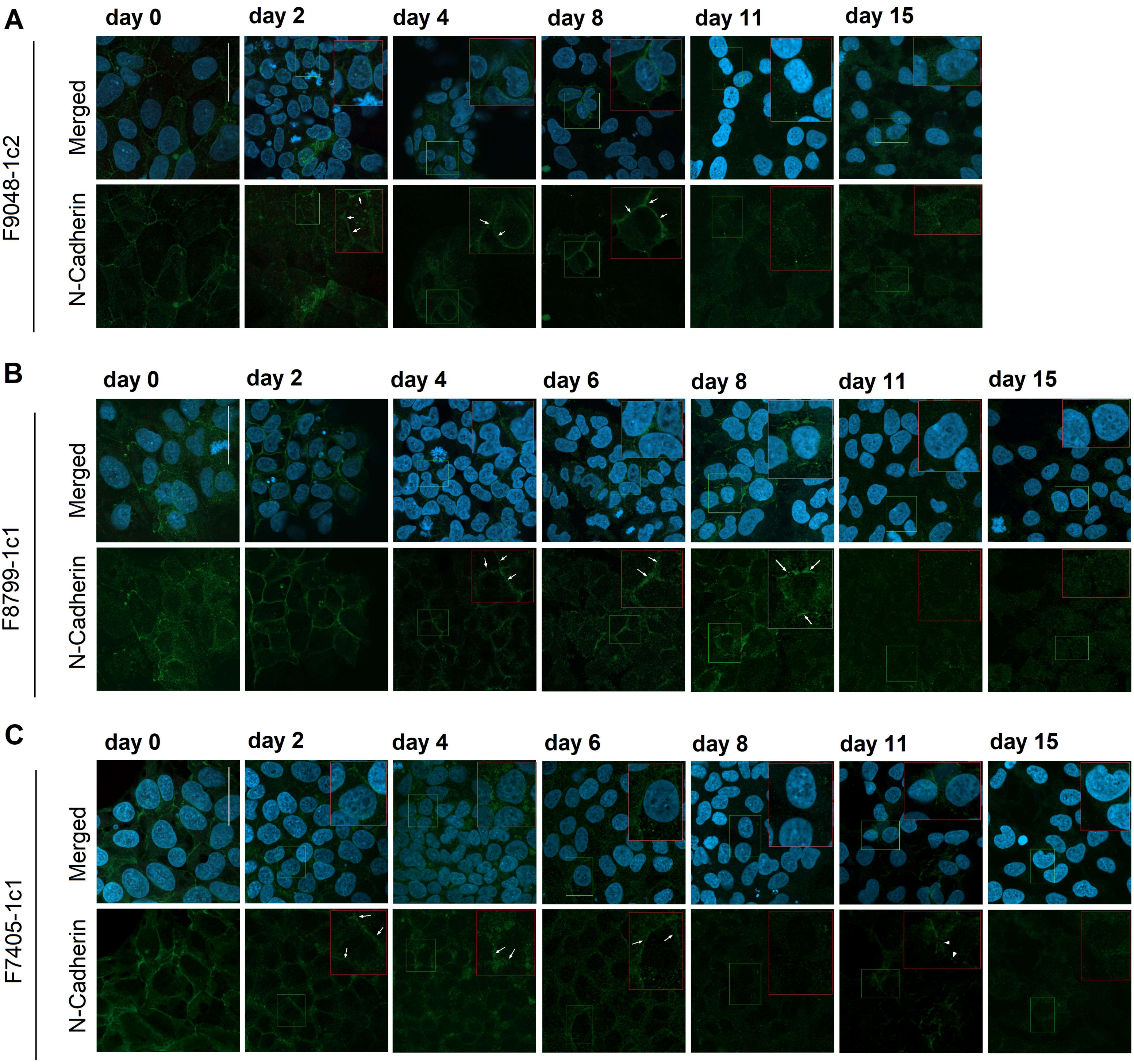
A
B
C
F9048-1c2
F8799-1c1
F7405-1c1
Merged
N-Cadherin
day 0
day 2
day 4
day 6
day 8
day 11
day 15

**Figure S3 (Related to Figure 3):** Time-series immunofluorescence analysis showing expression patterns for N-cadherin (CDH2) during NBC-to-NCC differentiation in all samples. Arrows indicate N-cadherin expression at cell-cell junctions. Arrowheads indicate membrane patches with diffuse N-cadherin expression. Scale bars: 45 µm. Insets: 2x magnification.

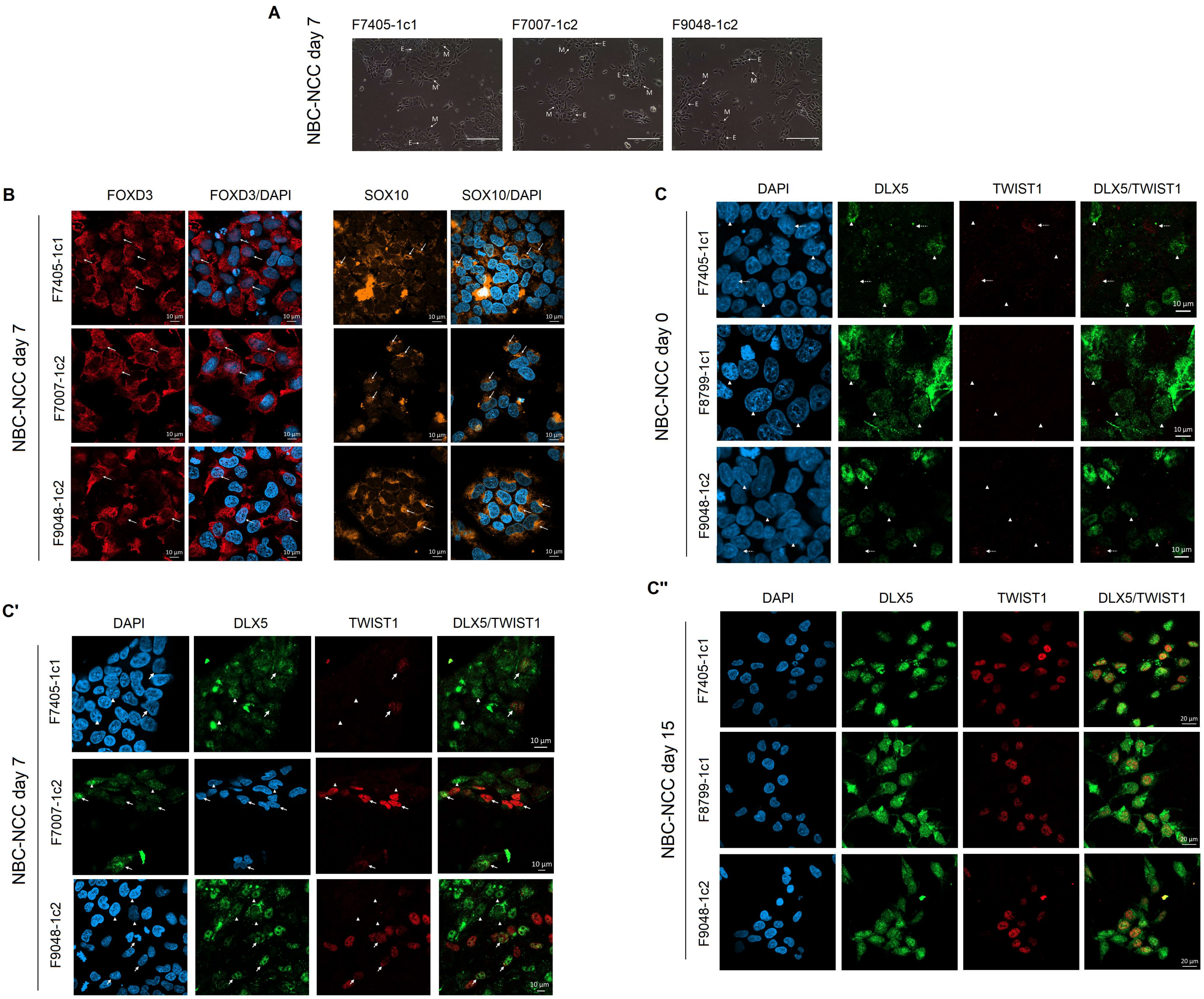
A
NBC-NCC day 7
F7405-1c1
F7007-1c2
F9048-1c2
B
FOXD3
FOXD3/DAPI
SOX10
SOX10/DAPI
NBC-NCC day 7
F7405-1c1
F7007-1c2
F9048-1c2
C
DAPI
DLX5
TWIST1
DLX5/TWIST1
NBC-NCC day 0
F7405-1c1
F8799-1c1
F9048-1c2
C'
DAPI
DLX5
TWIST1
DLX5/TWIST1
NBC-NCC day 7
F7405-1c1
F7007-1c2
F9048-1c2
C''
DAPI
DLX5
TWIST1
DLX5/TWIST1
NBC-NCC day 15
F7405-1c1
F8799-1c1
F9048-1c2

**Figure S4 (Related to Figure 4): A)** Phase-contrast micrographs depicting morphology of NBC-NCCs on day 7. E=epithelial-like clusters; M=mesenchymal-like cells. Scale bar: 200 µm. **B)** Immunostaining for NC markers FOXD3 and SOX10 in day-7 NBC-NCCs. Arrows point to nuclear expression (FOXD3) and perinuclear aggregates (SOX10). **C)** Double immunostaining analysis for DLX5xTWIST1 in NBC-NCCs on day 0; arrowheads point to DLX5$^{+}$cells; dashed arrows point to TWIST1$^{+}$ cells. **C', C'')** Double immunostaining for DLX5xTWIST1 was performed on NBC-NCC day 7 **(C')**, and day 15 **(C'')**. Arrowheads point to DLX5$^{+}$ cells; arrows point to DLX5$^{+}$/TWIST1$^{+}$ cells. In A, B and C', sample F8799-1c1 was lost and replaced by F7007-1c2 (see Experimental Procedures – Sample Setup). Scale bars: 10 µm (days 0/7); 20 µm (day 15).

Table S1: Primer sequences. Related to Experimental Procedures.

| Target | Forward primer (5'-3') | Reverse Primer (5'-3') |
|---|---|---|
| *ACTB* | TGAAGTGTGACGTGGACATC | GGAGGAGCAATGATCTTGAT |
| *CDH1* | CCATTCAGTACAACGCCCAACCC | CACAGTCACACACGCTGACCTC |
| *CDH11* | AGAGGTCCAATGTGGGAACG | GGTTGTCCTTCGAGGATACTGT |
| *CDH2* | TGCGGTACAGTGTAACTGGG | GAAACCGGGCTATCTGCTCG |
| *CDH6* | AGCTGCAGTTTCAGCCGCGA | AGGGTATCTCTGCTCGCCTTCC |
| *CDH7* | TCAAATACATCTTGTCAGGCGAA | TGGCATGAATATCCCCAGTGT |
| *CKIP1* | TCTGGAACTTGTCGGGTTGG | AGGTGGGGCAATAAGGGTTG |
| *c-kit* | ACGCACCTGCTGAAATGTATG | GTCTACCACGGGCTTCTGTC |
| *DLX5* | ACCAGCCAGAGAAAGAAGTGAC | CCTTCTCTGTAATGCGGCCA |
| *ETS1* | TCAAGGACTATGTGCGGGAC | TTGGTCCACTGCCTGTGTAG |
| *FOXD3* | ATTTCCTTTCCCCTGAGCCC | TTCGGTTTTCGGTTTTACCTG |
| *GAPDH* | ATCACCATCTTCCAGGAGCG | GGGCAGAGATGATGACCCTTT |
| *HMBS* | AGCTTGCTCGCATACAGACG | AGCTCCTTGGTAAACAGGCTT |
| *HPRT1* | CCTGGCGTCGTGATTAGTGAT | AGACGTTCAGTCCTGTCCATAA |
| *MITF* | AAATACGTTGCCTGTCTCGG | AGCTCCCTTTTTATGTTGGGAAG |
| *MSX2* | CACCCTGAGGAAACACAAGAC | TGCACGCTCTGCAATGGAG |
| *NANOG* | TGGACACTGGCTGAATCCTTC | CGTTGATTAGGCTCCAACCAT |
| *NGFR (P75)* | ACATAGCCTTCAAGAGGTGGA | TGTCGCTGTGGAGTTTTTCT |
| *OCT3/4* | GTGGTCAGCCAACTCGTCA | CCAAAAACCCTGGCACAAACT |
| *PAX3* | AAGCCCAAGCAGGTGACAAC | CTCGGATTTCCCAGCTGAAC |
| *PAX6* | TGTCCAACGGATGTGTGAG | TTTCCCAAGCAAAGATGGAC |
| *PRICKLE1* | TGGGGAACATATTGGTGTGGA | AGGCTTTACACTGGGCACAA |
| *SIX1* | TGGTTTAAGAACCGGAGGCA | GTTATTGTTTTCGGTGTTCTCCCTT |
| *SMURF1* | TTGAGGAGTCTTACCGCCAG | GGCACAGCAAGTAAAGCCAC |
| *SNAI2* | TCTGCGGCAAGGCGTTTTCCAG | GCAAATGCTCTGTTGCAGTGAGGG |
| *SOX10* | GCCTTACCACTCCTATGACTCC | TCAAAGCTACTCTCAGCCCC |
| *SOX17* | GGACCGCACGGAATTTGAAC | GGACACCACCGAGGAAATGG |
| *SOX2* | ATGTCCCAGCACTACCAGAGC | TTACTCTCCTCTTTTGCACCCC |
| *SOX9* | AGCGAACGCACATCAAGAC | CTGTAGGCGATCTGTTGGGG |
| *SP5* | TCGTGTGCAACTGGCTCTTCT | TTATTCTGGTGAGTCTTGACGTGC |
| *T* | ATGATGGAGGAACCCGGAGAC | GTACTGACTGGAGCTGGTAGG |
| *TBP* | GTGACCCAGCATCACTGTTTC | GCAAACCAGAAACCCTTGCG |
| *TFAP2A* | CTCCGCCATCCCTATTAACAAG | GACCCGGAACTGAACAGAAGA |
| *TWIST1* | CAATGACATCTAGGTCTCCGGGCCC | TACGCCTTCTCGGTCTGGAGGATG |
| *VIM* | GACAACCTGGCCGAGGACATCATG | AGACGTGCCAGAGACGCATTGTC |
| *ZIC1* | AAGGTCCACGAATCCTCCTC | TTGTGGTCGGGTTGTCTG |

**Table S2:** Antibodies used in this study. Related to Experimental Procedures.

| Antibody | Assays | Cell types | Identifier | Manufacturer |
|---|---|---|---|---|
| CD29-PE conjugate | Flow Cytometry | Ectomesenchymal stem cells | 555443 | BD Pharmingen |
| CD73-APC conjugate | Flow Cytometry | Ectomesenchymal stem cells | 560847 | BD Pharmingen |
| CD166-PE conjugate | Flow Cytometry | Ectomesenchymal stem cells | 559263 | BD Pharmingen |
| CD90-PE conjugate | Flow Cytometry | Ectomesenchymal stem cells | 555596 | BD Pharmingen |
| CD31-FITC conjugate | Flow Cytometry | Ectomesenchymal stem cells | 555445 | BD Pharmingen |
| CD45-FITC conjugate | Flow Cytometry | Ectomesenchymal stem cells | 347463 | BD Pharmingen |
| FITC-Mouse IgG1 K Isotype control | Flow Cytometry | Ectomesenchymal stem cells | 555748 | BD Pharmingen |
| APC-Mouse IgG1 K Isotype Control | Flow Cytometry | Ectomesenchymal stem cells | 554681 | BD Pharmingen |
| PE-Mouse IgG1 K Isotype control | Flow Cytometry | Ectomesenchymal stem cells | 555749 | BD Pharmingen |
| p75-Alexa Fluor 647 conjugate | Flow Cytometry | NBC-NCCs | 560877 | BD Pharmingen |
| HNK-1-FITC conjugate | Flow Cytometry | NBC-NCCs | 561906 | BD Pharmingen |
| FITC- Mouse IgM K isotype control | Flow Cytometry | NBC-NCCs | 555583 | BD Pharmingen |
| Alexa Fluor 647-Mouse IgG1 K isotype control | Flow Cytometry | NBC-NCCs | 557714 | BD Pharmingen |
| FOXD3 | Immunofluorescence | NBC-NCCs | orb69353 | Biorbyt |
| SNAI2 (SLUG) | Immunofluorescence | NBC-NCCs | #9585 | Cell Signaling |
| SOX10 | Immunofluorescence | NBC-NCCs, Schwann precursors | #89356 | Cell Signaling |
| AP2A | Flow Cytometry, Immunoflurorescence | NBCs | 10R-1283 | Fitzgerald |
| SOX1 | Flow Cytometry | NBCs | #4194 | Cell Signaling |
| PAX6 | Flow Cytometry, Immunoflurorescence | NBCs, NBC-NCCs | 42 6600 | Thermo |
| ZEB1 | Flow Cytometry, Immunoflurorescence | NBCs, NBC-NCCs | ab203829 | Abcam |
| DLX5 | Immunofluorescence | NBCs, NBC-NCCs | 70R-49665 | Fitzgerald |
| E-cadherin | Immunofluorescence | NBCs, NBC-NCCs | ab1416 | Abcam |
| N-cadherin | Immunofluorescence | NBCs, NBC-NCCs | ab19348 | Abcam |
| TWIST1 | Flow Cytometry, Immunoflurorescence | NBCs, NBC-NCCs | 10R-1347 | Fitzgerald |
| Peripherin | Immunofluorescence | Peripheral neurons | MA1-10034 | Thermo |
| βIII-tubulin | Immunofluorescence | Peripheral neurons | ab7751 | Abcam |
| S100B | Immunofluorescence | Schwann precursors | SAB1402349 | Sigma |
| Vimentin | Immunofluorescence | Smooth muscle | ab20346 | Abcam |
| αSMA | Immunofluorescence | Smooth muscle | ab5694 | Abcam |
| Musashi-1 | Immunofluorescence | NBCs | ab52865 | Abcam |
| Secondary Goat anti-Mouse IgG (H+L) Antibody, Alexa Fluor 488 | Immunofluorescence | - | A11001 | Thermo |
| Secondary Goat anti-Rabbit IgG (H+L) Antibody, Cyanine3 | Immunofluorescence | - | A10520 | Thermo |
| Secondary Goat anti-Rabbit IgG (H+L) C Antibody, Alexa Fluor 488 | Flow Cytometry, Immunoflurorescence | - | A11008 | Thermo |
| Secondary Goat anti-Mouse IgG (H+L) Antibody, Alexa Fluor 647 | Immunofluorescence | - | A21235 | Thermo |
